# Can amniotic fluid protect developing fetal lungs against the harmful effects of oxidative stress?

**DOI:** 10.55730/1300-0144.5564

**Published:** 2022-09-18

**Authors:** Levent KORKMAZ, Cumali ALAN, İsmail TOPAL, Mahir TAYFUR, Ali Seydi BOZKURT, Cebrail GÜRSUL, Osman BAŞTUĞ

**Affiliations:** 1 Division of Neonatology, Department of Pediatrics, Faculty of Medicine, Erzincan Binali Yıldırım University, Erzincan, Turkey; 2 Department of Pediatrics, Faculty of Medicine, Erzincan Binali Yıldırım University, Erzincan, Turkey; 3 Department of Pathology, Faculty of Medicine, Erzincan Binali Yıldırım University, Erzincan, Turkey; 4 Department of Urology, Faculty of Medicine, Erzincan Binali Yıldırım University, Erzincan, Turkey; 5 Department of Physiology, Faculty of Medicine, Erzincan Binali Yıldırım University, Erzincan, Turkey; 6 Division of Neonatology, Department of Pediatrics, Kayseri Training and Research Hospital, Kayseri, Turkey

**Keywords:** Amniotic fluid, hyperbaric oxygen, oxidative stress, bronchopulmonary dysplasia

## Abstract

**Background/aim:**

Preterm births cause fetuses to be born without completing the development of their organs. Due to this undesirable situation, it is the pulmonary tissue which has to be most exposed to harmful effects of extrauterine environment. Early disappearance of the prophylactic and constructive effects of amniotic fluid (AF) on developing tissues, such as pulmonary tissue, facilitates the formation of pulmonary morbidities resulting from oxygen. Setting out from this knowledge, we wanted, in addition to assessing the beneficent effects of AF on pulmonary tissue, to study the importance of AF in morbidities of this tissue thought to originate from oxygen.

**Materials and methods:**

In this experimental study, while the study group was made up of the fetuses of pregnant rats exposed to hyperbaric oxygen, (hyperoxic pregnant rat fetuses-HPRF), the control group was formed of the fetuses of the rats pregnant in the usual room setting (normoxic pregnant rat fetuses-NPRF). The pulmonary and hepatic tissues taken from the fetuses of these pregnant rats on the 21st day of their pregnancy were compared biochemically and histologically. For biochemical assessment, total glutathione (tGSH), catalase (CAT), malondialdehyde (MDA), tumor necrosis factor-alpha (TNF-α) values and for histopathological assessment, apoptosis, alveolar wall count (AWC), vena centralis count (VCC) were included.

**Results:**

Statistical significance was found in the pulmonary tissue values of tGSH on behalf of NPRF, and MDA on behalf of HPRF (p < 0.05). In liver tissue, statistical significance was detected in tGSH and CAT values in favor of NPRF and in MDA, and TNF-α values in favor of HPRF (p < 0.05).

**Conclusion:**

Our study has demonstrated that AF protects the pulmonary tissue from the harmful effects of oxygen in the intrauterine period. In addition, our data have suggested that the pulmonary tissue’s being deprived of the useful effects of AF owing to premature birth may be an important trigger in the occurrence of the pulmonary morbidities thought to result from oxygen.

## 1. Introduction

Intrauterine fetuses can maintain their nourishment and development ideally, away from the harmful effects of extrauterine environment. However, the fetus moving out of this ideal setting, owing to premature birth, loses its comfort and has to face the problems of extrauterine setting for which it is not ready yet. The most important of these problems is oxygen, which is at a level higher than in intrauterine setting. Normally, reactive oxygen species (ROS) which form during the aerobic metabolism of the cell is neutralized by the antioxidant (AOx) systems of the organism. Yet, in some cases, ROS production exceeds the capacity of AOx defense system. Since preterm newborns, in particular, have immature AOx system, they are more prone to oxidant stress (OxS) than other newborns [1–3].

In the intrauterine period, low arterial oxygen pressure (PaO_2_) keeps the OxS level restricted. Following the birth, with the start of spontaneous respiration, PaO_2_ rises rapidly to the extrauterine level. This change results in a rapid change in OxS [3–5]. The preterm newborns with immature AOx systems become, in the early period, deprived of the beneficial effects of AF, which balances OxS and enables tissue development, in addition to having to face the elevated OxS in extrauterine setting, in the same early period. If the increase in OxS cannot neutralize for some reason, lipid peroxidation and cellular damage starts in the tissue. Once triggered, this reaction increases on its own and continues progressively until it is stopped by AOx [3,6–10]. As a result of this vicious circle, important morbidities such as bronchopulmonary dysplasia (BPD), necrotising enterocolitis (NEC), retinopathy of prematurity (ROP), intraventricular hemorrhage (IVH), periventricular leukomalacia (PVL) are triggered [8,11–13].

In addition to all these, it is also known that, owing to placental mitochondrial activity which has been elevated in a normal pregnancy period, the OxS in the intraamniotic setting also increases physiologically. In fact, the increase in OxS is essential for pregnancy to continue. Nonetheless, this intraamniotic setting is neutralized by the increasing OxS and AOx systems and hence OxS-AOx balance is achieved begetting a setting where the fetus can maintain its development in ideal conditions [6,7,14,15]. Amniotic fluid (AF), which is considerably important in providing this setting in question, is rich in cells and mediators such as amniotic fluid stem cells (AFSC), amniotic fluid mesenchymal stem cell (AFMSC), insulin-like growth factor (IGF)-1, vascular endothelial growth factor (VEGF), fibroblast growth factor (FGF), epidermal growth factor (EGF), platelet derived growth factor (PDGF), transforming growth factor alpha/beta (TGF-α/β), granulocyte-colony stimulating factor (G-CSF), placental growth factor (PIGF), angiopoietin (Ang)-1, hyaluronic acid, platelet activating factor acylhydrolase and platelet activating factor acetyl transferase. Fetal pulmonary development is achieved through the interaction, between themselves in balance, of these mediators and growth factors in AF [3,16–23].

AF contains cells such as AFSC and AFMSC, and the beneficial effects of these cells on lung tissue are also reported in studies in the literature. In addition, it has been shown that these cells can differentiate into different cell types of various tissues, especially lung tissue [18,24–26]. In addition to all these, it has been shown that these cells reduce the damage to the lung tissue against the harmful effects of hyperbaric oxygen (HBO) and increase tissue regeneration. It has also been shown that these cells exert their beneficial effects by increasing immune modulation, angiogenesis and decreasing apoptosis [18,26–31].

Even in today’s modern neonatal intensive care unit (NICU) capabilities, with the prophylactic interventions in the foreground, such as advanced methods of mechanical ventilation, surfactant applications, restriction of oxygen use and prenatal steroid treatments, it is known that BPD is still encountered frequently as new BPD [2,3,11]. This suggests the idea that new BPD may, in fact, be a clinical result of the early interruption of the abovementioned helpful effects of AF. This notion raises the thesis that there might be a more important predisposing factor than oxygen in the occurrence of morbidities thought to depend on oxygen like BPD.

In order to investigate our thoughts which had shaped in line with this literature knowledge and to be able to find logical answers to the questions regarding this subject, we formed hyperoxia in the fetuses of pregnant rats having immature AOx system and and aimed to understand the protectiveness of AF in lung tissue. Thus, we wanted to assess not only the tissue improving and prophylactic effect of AF against the harms of oxygen on pulmonary tissue but also the role of AF in morbidities thought to stem from oxygen.

## 2. Materials and methods

Consent for our study planned as an experimental animal study was obtained from Atatürk University Animal Ethics Committee (Resolution No: 26.12.2020-264). Sexually mature female Wistar rats were obtained from the Experimental and Clinical Research Center and made pregnant. The pregnant rats were allowed to have ad libitum access to pellet food and water. The room temperature was kept constant at 23 ± 1ºC and the 12-hour night-day cycle was observed. The work started on 30.12.2020 and was completed on 25.06.22021. All the animals were treated in accordance with the Regulation of the Usage and Care of Laboratory Animals.

**Figure 1:**
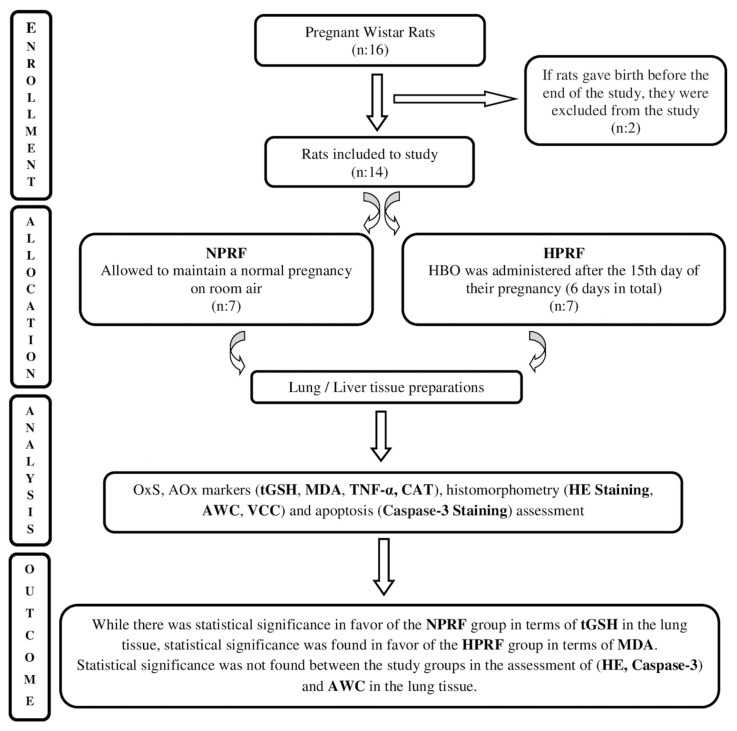
Study groups and experimental design. **NPRF**: normoxic pregnant rat fetuses; **HPRF:** hyperoxic pregnant rat fetuses; **HBO**: hyperbaric oxygen; **OxS**: oxidant stress; **AOx**: antioxidant; **tGSH**: tissue total glutathione; **MDA**: tissue malondialdehyde; **TNF-α**: tissue tumor necrosis factor-alpha; **CAT**: tissue catalase; **HE**: hematoxylin and eosin; **Caspase-3**: cysteine aspartyl proteases; **AWC**: alveolar wall count; **VCC**: vena centralis count.

The groups for the study were composed of the intrauterine period fetuses of these pregnant rats. The control group consisted of the fetuses of the pregnant rats sustaining their pregnancy in the ordinary room setting (normoxic pregnant rat fetuses-NPRF), and the fetuses of the pregnant rats subjected to HBO constituted the study group (hyperoxic pregnant rat fetuses-HPRF) (Figure 1).

The pregnancy of 16 female rats in total was achieved. The routine pregnancy follow-ups were done by an experienced veterinary physician. Seven of these pregnant rats underwent a daily 2-hour HBO application for 6 days beginning on the 15th gestational day (HPRF-study group). HBO application was carried out in a completely closed metal container capable of providing the externally controllable targeted pressure. A maximum of 2 rats were allowed in this container each time. During the application, while the mothers’ nutritional support was maintained, hyperbaric oxygen 2.8 atmospheric pressure with 100% O_2_ inhalation was administered (30). The conditions necessary for the 7 remaining pregnant rats to maintain a normal pregnancy by allowing them to inhale room air alone (NPRF-control group).

HPRF pregnant rats were put to sleep in HBO container on the 21st gestational day at the suggestion of the veterinary physician. The lungs and liver of the most suitable one of the fetuses of these mothers were taken and thus the HPRF group was formed of these tissues. The pregnant rats in the room setting were put to sleep and most suitable fetus lung/liver was removed for the NPRF group to be formed. While the groups in question were being formed, the pulmonary and hepatic tissues of only one fetus of each mother were taken. One of the lungs taken was used for biochemical assessment and the other for histopathological (histomorphometry, apoptosis) assessment. If any of the mother rats had an early delivery, such baby rats were not included in the study. Each tissue has a different AOx response to OxS, in terms of AOx capacity, the AOx capacity of liver tissue is higher than that of other tissues. For these reasons, we did not make a statistically comparison of liver and lung tissues in our study.

While the fetus tissues were being taken, i.p. heparin (50U) was administered to prevent fibrin accumulation and intraperitoneal Xylazine (80–100 mg/kg: 5–10 mg/kg) for anesthesia. The abdomen and thorax were opened 30 s later. The right lung was preserved at −70 ºC for the study of biochemical markers (Ox, AOx) while the left lung was preserved in formaldehyde solution for histomorphometry and apoptosis assessment.

### 2.1. Tissue total glutathione (tGSH) measurements

The tGSH in the tissue was measured according to the method of Sedlak and Lindsay [31]. The sample was weighed and homogenized in 2 mL of 50 mmol/L Tris-HCl buffer containing 20 mmol/L ethylenediamine tetraacetic acid and 0.2 mmol/L sucrose at pH = 7.5. The homogenate was precipitated with 0.1 mL of 25% trichloroacetic acid; the precipitate was removed after centrifugation at 4200 rpm for 40 min at 4 °C, and the supernatant was used to determine tGSH value. A total of 1500 μL of measurement buffer, 500 μL supernatant, 100 μL 5,5-dithiobis-(2-nitrobenzoic acid) (DTNB) (10 mmol/L) and 7900 μL methanol were added to a tube and then vortexed and incubated for 30 min at 37 °C. Used as a chromogen, DTNB formed a yellow-colored complex with sulfhydryl groups. Absorbance was measured at 412 nm, using a spectrophotometer (Beckman DU 500, USA). The standard curve was obtained with respect to reduced glutathione.

### 2.2. Tissue catalase (CAT) measurements

Decomposition of H_2_O_2_ in the presence of CAT was measured at 240 nm. The CAT level was defined as the amount of enzyme required to process 1 nM H_2_O_2_ per minute at 26 °C and pH 7.8.

### 2.3. Tissue malondialdehyde (MDA) measurements

MDA measurements were made according to the method defined by Ohkawa et al. [32]. The tissue homogenate sample (0.1 mL) was added to a solution containing 0.2 ml of 80 g/L sodium dodecyl sulfate, 1.5 mL of 200 g/L acetic acid, 1.5 mL of 8 g/L 2-thiobarbiturate, and 0.3 mL distilled water. The mixture was incubated at 95°C for 1 h. Upon cooling, 5 mL of n-butanol: pyridine (15:1) was added. The mixture was vortexed for 1 min and centrifuged for 30 min at 4000 rpm. The absorbance of the supernatant was measured at 532 nm.

### 2.4. Tissue tumor necrosis factor-alpha (TNF-α) measurements

TNF-α concentrations in the tissue homogenate were measured using the following rat-specific sandwich enzyme-linked immunosorbent assay kits: Rat TNF-α ELISA kits (Cat no: YHB1098Ra, Shanghai LZ). Analyzes were made taking into account the manufacturer’s instructions. The wells of the microplates were coated with the monoclonal antibody specific for rat TNF-α. The tissue homogenate biotinylated monoclonal antibody specific and streptavidin-HRP were pipetted onto the wells and incubated at 37 °C for 60 min. After, chromogen reagent A and chromogen reagent B were added, which are acted upon by the bound enzyme to produce a color. It was incubated at 37 °C for 10 min and the stop solution was added. The color intensity of this product is directly proportional to the concentration of rat TNF-α present in the original specimen. At the end of the procedure, the well plates were read at 450 nm using a microplate reader. The absorbance of the samples was estimated using formulas obtained from standard charts.

### 2.5. Hematoxylin and eosin (HE) staining methods and apoptosis count

Tissue samples were prepared from the liver and lung of rats. This tissue samples were fixed in formaldehyde for 24 h at room temperature for fixation. Later, closed system tissue processing method was applied to these tissue samples. Tissue samples were stored in 9 containers including various solutions at 30–45 °C and 3 paraffin container at 48–68 °C. These solutions and their duration in containers are as follows: formaldehyde 10% (2 h) (35 °C, vacuum), formaldehyde 1.5% (? h), formaldehyde 10% (1 h), alcohol %95 (1 h) (vacuum), alcohol %95 (1 h), absolute alcohol (45 min), absolute alcohol (1 h), xylene (1 h), xylene (1 h), paraffin (1/2 h), paraffin (1 h), paraffin (1 h). Tissue embedded paraffin blocks were prepared. Sections of 4 micron thickness were prepared from paraffin block embedded in each tissue, and then these sections were placed in hot water at 80 degrees. Later, they were taken on the slide and the deparaffinization was applied to them and were stained with HE staining. The distilled water was applied to the slides 10 min after staining with Hematoxylin for 5 min. The alcohol 95% was applied to the slides 10 min after staining with Eosin for 3 min. After they were kept on the staining slides, they were closed with a coverslip and kept for 30 min for drying.

### 2.6. Cysteine aspartyl proteases (caspase)-3 staining (immunohistochemical procedures) method and apoptosis count

For immunohistochemical (IHC) staining, primary antibodies of caspase-3 antibody (Santa Cruz Biotechnology, Dallas, TX: 1/100 and Cell Signaling Technology Inc., Danvers, MA) were used. Use was made of an automatic IHC device (Leica Bond-Max, Australia) to stain the sections. Having been processed in IHC device, the sections were dehydrated through a graded series of ethanol to xylene and closed (Entellan, Merck Millipore, Germany). For IHC operation, sections with a thickness of 4 μm were formed on a positively charged microscope slides were formed of the tissue samples in 10% formalin solution. The results of the analysis performed with Olympus BX51 microscope were assessed with caspase-3 staining of the tissues.

### 2.7. Quantification method of apoptosis and histomorphometry in hepatic/pulmonary tissues

At the stage of histopathological study and grading, the number of the apoptotic cells in randomly selected 5 different sites on tissue preparate, which had been stained with HE and caspase, was counted (33). The sum of the total number of apoptosis was averaged. In histomorphometric assessment following the HE staining, grids were formed with evenly distributed parallel lines of 200 μm long and 65 μm apart on the 10-fold magnified pulmonary section taken from study station. Alveolar walls intersecting throughout the grids which reflect the extent of alveolarization and pulmonary development were marked and counted (green points, alveolar wall count -AWC). While counting, blood vessels, bronchi, and bronchioles were excluded. Foe each preparate, AWC on 10 lines were assessed and for each preparate the mean AWC value per line was calculated. In the study of hepatic histomorphometry, the vena centralis count (VCC) in the hepatic lobule center in randomly selected five locations (×200) of each preparate was counted and the mean VCC per preparate was included in statistical analysis (34,35).

### 2.8. Statistical analysis

The SPSS 21.0 (IBM Corporation, Armonk, NY, USA) program for statistical analyses was used. The data obtained from the study were transferred to electronic environment and analyzed in this statistical program. The descriptive values of the data obtained from assessment and analyses were presented as mean, standard deviation (SD) depending on the types of their features. Use was made of Shapiro-Wilk test to determine whether or not the data had a normal distribution. Normally distributed data were compared by means of t test. In the intergroup comparison of the data without normal distribution, Mann-Whitney U test was used. p < 0.05 was taken as point of statistical significance.

## 3. Results

**Figure 2:**
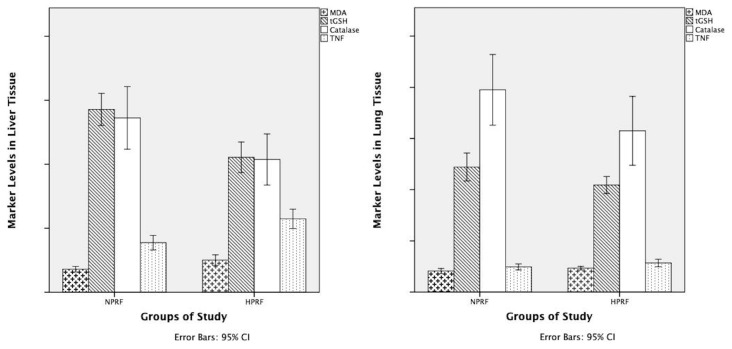
Oxidant and antioxidant marker levels in lung tissue. **NPRF**: normoxic pregnant rat fetuses; **HPRF**: hyperoxic pregnant rat fetuses; **MDA**: malondialdehyde (pmol/ml); **tGSH**: total glutathione (nmol/mL); **CAT**: catalase (U/mL); **TNF-a**: tumor necrosis factor-alpha (pg/mL).

**Figure 3:**
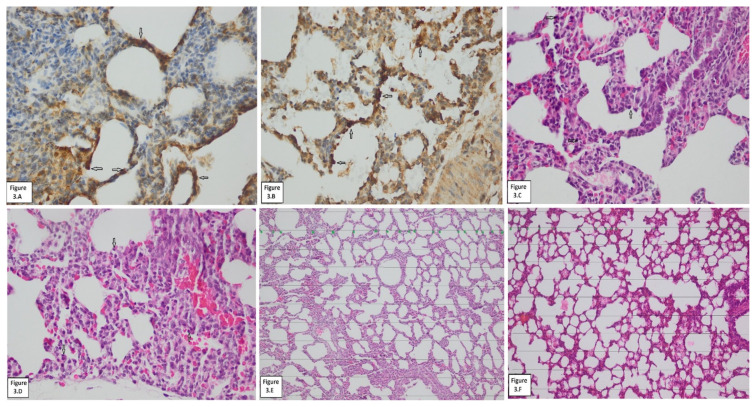
Hematoxylin eosin-caspase staining of lung tissues. **(A)** apoptotic cells stained by caspase method in lung tissue of the NPRF group (white arrows); **(B)** apoptotic cells stained by caspase method in lung tissue of the HPRF group (white arrows); **(C)** apoptotic cells stained by HE method in lung tissue of the NPRF group (white arrows); **(D)** apoptotic cells stained by HE method in lung tissue of the HPRF group (white arrows); **(E)** AWC stained by HE method in lung tissue of the NPRF group (spots); **(F)** AWC stained by HE method in lung tissue of the HPRF group (spots). **NPRF**: normoxic pregnant rat fetuses; **HPRF**: hyperoxic pregnant rat fetuses; **HE**: hematoxylin eosin; **AWC**: alveolar wall count.

**Figure 4:**
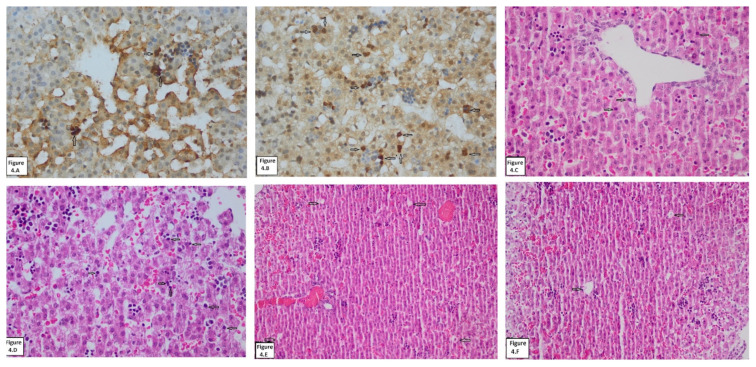
Hematoxylin eosin-caspase staining of liver tissues. **(A)** apoptotic cells stained by caspase method in liver tissue of the NPRF group (white arrows); **(B)** apoptotic cells stained by caspase method in liver tissue of the HPRF group (white arrows); **(C)** apoptotic cells stained by HE method in liver tissue of the NPRF group (white arrows); **(D)** apoptotic cells stained by HE method in liver tissue of the HPRF group (white arrows); **(E)** VCC stained by HE method in liver tissue of the NPRF group (white arrows); **(F)** VCC stained by HE method in liver tissue of the HPRF group (white arrows). **NPRF**: normoxic pregnant rat fetuses; **HPRF**: hyperoxic pregnant rat fetuses; **HE**: hematoxylin eosin; **VCC**: vena centralis count.

In our study, in which we aimed to understand the effects of AF on tissue growth in immature pulmonary and hepatic tissue, while our OxS and AOx markers data helped us understand the changes in tissue level, our histopathology (histomorphometry, apoptosis) data helped us understand the clinical reflections AF in addition to its AOx effects. The data regarding the OxS and AOx markers in the hepatic and pulmonary tissues of our study have been presented in Table 1 and Figure 2, and histopathology and histomorphometry in Table 2 and Figures 3A–3F and 4A–4F.

The prepregnant weights of all the mother rats in all study groups were in the range of 190–220 g and the mean birth weight of the fetal rats was 4.5 ± 0.3 g. We started the study with 16 female Wistar rats. Two of the pregnant rats were excluded from the study because they gave birth naturally. As a result, we completed our study with 14 pregnant Wistar rats. Statistical significance was found in favor of NPRF cases in tGSH (12.22 ± 1.64, 10.46 ± 0.99) data and in MDA (2.05 ± 0.31, 2.34 ± 0.21) data in the fetal pulmonary tissues in favor of HPRF cases (Table 1; Figure 2) (p < 0.05). Statistical significance was not found in CAT and TNF-α data in this tissue between the study groups (Table 1; Figures 2 and 3A–3F) (p > 0.05). In apoptosis (HE, caspase) and histomorphometry (AWC) assessment on pulmonary tissue, however, statistical significance was not found between the study groups (Table 2; Figures 3A–3F) (p > 0.05).

As for the hepatic tissue in our study, statistical significance was detected in all the biochemical data showing OxS, AOx markers. Statistical significance was found in tGSH (14.29 ± 1.50, 10.54 ± 1.44) and CAT (13.63 ± 2.92, 10.38 ± 2.39) data in favor of NPRF and in MDA (1.81 ± 0.26, 2.52 ± 0.49), and TNF-α (3.88 ± 0.68, 5.74 ± 0.91) data in favor of HPRF (Table 1; Figure 2) (p < 0.05). In hepatic tissue, tGSH, CAT values which show the effect of AOx were found low in HPRF cases (10.54 ± 1.44, 10.38 ± 2.39) while MDA, TNF-α values (2.52 ± 0.49, 5.74 ± 0.91) which show the effect of OxS were found high in the same tissue (p < 0.05).

In the apoptosis (HE, caspase) assessment on the hepatic tissue of the study groups, it was found that there existed data indicating statistical significance (12.2 ± 2.14, 3.83 ± 1.04) in favor of HPRF (12.2 ± 2.14, 3.83 ± 1.04) (Table 2; Figures 4A–4D) (p < 0.05). In histometry (VCC) assessment, however, statistical significance was not detected in any of the groups (Table 2; Figures 4E and 4F) (p > 0.05).

## 4. Discussion

This study has shown that AF protects pulmonary tissue from the toxic effects of oxygen and contributes to the sustenance of normal pulmonary tissue growth more than it is thought. Furthermore, our data have suggested that this tissue becomes vulnerable to the harmful factors in the external environment as a result of premature birth, that the mechanisms impairing tissue growth mobilize in early period and that the physiopathology responsible for the formation of oxygen-dependent morbidities are triggered in this period. Looking at the literature data on the subject, it is noticed that AF is rich in cells and mediators such as AFSC, AFMSC, IGF-1, VEGF, G-CSF, PIGF, Ang-1, FGF, EGF, PDGF, TGF-α/β, hyaluronic acid, platelet activating factor (PAF) acylhydrolase, PAF acetyltransferase. In addition to transporting to the fetus in the intrauterine period the mediators necessary for it to growth, AF provides the ideal ambience for these factors to interact with each other as a form of balance. Based on all these literature data, our study showed that the real predisposing factor in pulmonary morbidities is thought to be responsible is the early interruption of the beneficial effects of AF, rather than oxygen.

Fetal life is maintained in a relatively hypoxic ambience compared to intrauterine life. Therefore, in utero PaO_2_ is rather low compared to that of mother. Immediately after birth, with spontaneous respiration beginning, PaO_2_ reaches its extrauterine level in a short time [3–6,8,32]. The increasing PaO_2_ cause physiologic ROS to increase and trigger the expression of a series of important genes which encode AOx system. This is a physiological process that renders the organism amenable extrauterine pulmonary respiration [8–10]. However, preterm newborns cannot neutralize the increase in ROS since they have immature AOx and immature mitochondrial systems. It is known that AOx enzyme activity runs low until the last period of pregnancy and can show a dramatic increase as much as 500% at the end of pregnancy. Therefore, the premature newborns’ responses to hypoxia is inadequate due to their low capacity of AOx. Consequently, the ROS, which cannot be neutralized, activates apoptosis and proinflammatory mediators [1,3,8,9,33–38].

There are many different cells and mediators in AF. Therefore, many researchers are currently testing AF treatments in experimental studies. The researchers focused on the lung tissue in a significant part of these studies and reported the beneficial effects of AF on this tissue. In parallel with this information, it has been proven today that AFSCs can differentiate into different cell types and that these cells can participate in the regeneration of various tissues, especially lung tissue. AFMSCs are particularly interesting because of their potential benefits when considering them for medical use. AF is a novel stem cell source that is regarded as medical waste. Interestingly, it has been shown that tissue damage is reduced and tissue regeneration increased when AFMSCs were transplanted with HBO administration. AFMSCs, such as AFSCs, have been reported to have potent beneficial effects on lung tissue regeneration. These cells have been shown to exert their beneficial effects by increasing immune modulation and angiogenesis and decreasing apoptosis [18,24–26].

Though postpartum increase in OxS occurs in all tissues, in this regard, pulmonary tissue has a separate place from other tissues. It is probably the pulmonary tissue which most benefits from the useful effects of this fluid balancing OxS and providing tissue growth due to the beneficial cells for tissue development it contains in the intrauterine period. However, this advantage of the lungs in the intrauterine period converts slowly into disadvantage owing to preterm birth. It is because this tissue, growing in an ideal environment in the intrauterine period, changes into one, as a result of preterm birth, which has maximum contact with oxygen and thus has to face the destructive effects of oxygen.

In our study we have determined that AF protects the pulmonary tissue of fetuses more effectually against the harmful effects of hyperoxia than it does hepatic tissue. Our data have indicated that hyperoxia does not increase apoptosis symptoms in the lungs but increases apoptosis in the liver in a way compatible with all biochemical data. Absence of difference in apoptosis and AWC data in the lungs between the groups was indicative of the protection of this tissue by AF. In the liver, however, this increase in favor of HPRF in apoptosis data indicates that this tissue cannot be protected against hyporexia by AF while the absence of any statistical difference in VCC data between the groups indicates that it would be appropriate to make more studies on the subject with more samples.

The damage and pulmonary cell proliferation that occur in the lungs of the rats exposed to 60% FiO_2,_ instead of 95% cannot entirely be stopped. In line with this, there are studies in the literature reporting that the relationship between oxygen use and BPD is weak [2,18,39–42]. In addition, in infants at risk of developing BPD, reduction of exposure to oxygen, even permissive hypercapnia application has failed to bring about a considerable decrease in BPD. The BPD type generally born earlier than 28 weeks without RDS symptoms and not requiring mechanical ventilation or oxygen support is called new BPD in our time and this form constitutes a great majority of the patient population. New BPD is known to emerge as a result of the disruption of the intrauterine growth of the pulmonary tissue and the postpartum exposure of the lungs to the harmful extrauterine factors. It must also be kept in mind that AF is rich in mediators which enable normal tissue growth to continue in addition to its prophylactic effect again OxS [3,36].

In the literature on the studies done as randomized clinical trials and in metaanalyses it has been reported that the practice of noninvasive respiratory support does not reduce BPD as much as expected compared to mechanical ventilation [2]. In addition to all these new observations, although in our present day NICU’s sensitive mechanical ventilation methods and systems that provide controlled oxygen support are used, underlying causes of encountering BPD as new BPD still at higher rates have been something of an enigma for researchers. The researchers seeking answer to this question have drawn attention to anatomic and pathophysiologic similarities between the placenta and the lungs, and propounded that the cause of these high rates may be the placental dysfunction in newborns concerned [11,43]. However, in studies regarding the subject, the direct relation between the developmental differences of placenta and fetal pulmonary tissue growth has not been able to be shown precisely and it has been thought that there may be more different factors effective on pulmonary tissue development [43,44].

When all this literature is taken into consideration, the thought in researchers’ minds that a more important and more critical factor than both oxygen and respiratory support plays a role in the development of BPD gains importance. In our study we reached the data not quite mentioned in the relevant literature but showing that early cutoff the useful effects of AF on pulmonary tissue must be more important than it is thought. When looked from this perspective, our study, by presenting to the literature the data which enable us to question the place and importance of oxygen in the occurrence of morbidities resulting from oxygen, has provided a different and comprehensive approach to the questions involved about the pathophysiology of BPD.

In the literature there are some discrepancies in the assessment of OxS, AOx markers. That the AOx markers increase and OxS markers decrease compared to the control group, or absence of difference between the groups is thought to indicate that AOx system is active and protects the organism from the harmful effects of OxS [14,39,45]. tGSH is the most abundant major intracellular AOx marker in the body. CAT also has recently been shown to affect the incidence of morbidity in premature babies. These two AOx markers constitute the most appropriate enzymatic defense against ROS. One of the last products of MDA lipid peroxidation, TNF-α, however, is measured as OxS indicator because it is the most important proinflammatory mediator [1,6,8,37–39]. Increasing of AOx markers and decreasing of OxS markers compared with normal tissue or absence of statistically significant differences between these markers indicate that the tissue is protected against OxS. Viewed from this perspective, CAT, TNF-α presented vales suggesting that AF protect the lungs against the harmful effects of OxS in contrast to tGSH, MDA values suggesting that no such exist. In addition, the results of our study included AOx, OxS marker and histopathology data of lung tissue, indications that AF protects this tissue from the damaging effects of OxS.

ROS production increases as a result of the placental mitochondrial activity and maternal metabolism that have increased in pregnancy. AF plays a vital role in the rapid neutralization of physiologic ROS increase in pregnancy by the AOx mechanisms similarly increasing. In fact, this controlled increase in OxS is necessary for pregnancy to continue. If this balance in pregnancy is upset in favor of OxS for reasons such as an increase in OxS or a decrease in AOx, both premature birth and the aforementioned morbidities caused by this birth are triggered [6,7,45]. When lipid peroxidation starts (increase in MDA, TNF-α) as a result of an increase in OxS, this injury proliferates on its own and this reaction continues until being stopped by an AOx. In the etiology of premature birth, the presence of similar mechanisms, such as an increase in OxS, responsible for the occurrence of preterm birth-related morbidities presents important clues to understand the pathophysiology of the morbidities in question [6,46–49].

When our study groups were compared in terms of hepatic tissue data, it was seen that tGSH and CAT, which are AOx markers in hyperoxia group, were decreased while MDA and TNF-α, which are OxS markers, were elevated. These data were clear indicators of the failure of AF to protect the hepatic tissue against hyperoxia. However, when our study groups are compared in terms of pulmonary tissue, the levels of CAT and TNF-α markers having remained unchanged indicates that AF will be able to protect the pulmonary tissue from OxS, the same situation is not in question in terms of MDA and tGSH markers. It is possible to think that this inconsistency may have resulted from the feedback mechanisms between the reactions and interactions of OxS – AOx. Given the absence of any finding in our data to suggest any prophylactic effect of AF on hepatic tissue, our study has revealed some clues about the contribution of AF, in the intrauterine period, to the maintenance of normal pulmonary tissue growth, by protecting the pulmonary tissue from the harmful factors. Given that the beneficial effects of AF on the lungs are impaired by premature birth, the idea that is raised is that this disruption may be effectual in the occurrence of morbidities such as new BPD, which have already started in intrauterine period, but triggered by OxS. When looked from this perspective, our study corroborates the relevant studies in the literature, on the one hand, and presents a perspective able to eliminate the confusion caused by BDP frequently encountered in our present day NICUs, on the other hand.

IGF-1 found abundantly in AF is both the basic mediator of inflammatory response and has a critical importance for the action of VEGF. Additionally, it has been demonstrated that AFSCs in AF are capable of differentiating into several cell types such as epithelial cells and rectifying pulmonary pathology considerably by improving the alveolarization of the treatment with AFSCs of the rat lungs exposed to hyperoxia, organization of capillary webs and angiogenesis [16–19,25,26,39,45,49–51].

In addition to EGF, which serves the maturation of the lungs and is present at high levels in AF, TGF-α has a structure similar to this mediator and binds the same receptor. The presence in high levels in AF, of PAF acylhydrolase and PAF acetyltransferase enzymes, the disintegrators of PAF, known to be a powerful vasoconstrictor and play a key role in a multitude of morbidities, have a prophylactic effect against serious diseases. Additionally, found abundantly in AF, hyaluronic acid and TGF-β, which impart to AF the quality to reduce scars by inhibiting collagen synthesis, are important in this prophylactic effect [3,16,17,20–23]. In addition to these factors, AF’s being rich in AOx mediators (IGF-1, VEGF, Ang-1) and developmental factors (G-CSF, PIGF, Ang-1, FGF, EGF, PDGF, TGF-α/β) has an important place in its prophylaxis against the harmful factors exposed to in the intrauterine period [18,19].

When looked at our apoptosis data, which enable us to understand the OxS induced structural changes in the tissues, absence of statistical difference between the study groups in terms of the apoptosis data in the pulmonary tissue, can be regarded as another indicator of the prophylactic effect of AF on pulmonary tissues. In our study no difference has been found between both apoptosis and AWC data in the hepatic tissue. From this point of view, it is absolutely reasonable to think that the AFSMCs and AFSCs contained in AF are effective on lung tissue development. Our study data have revealed that hyporexia triggers apoptosis in the hepatic tissue but does not cause any difference in VCC values [18,25,26]. More advanced cellular studies are required so that more consistent assumptions can be propounded on the subject of VCC values not being changed by hyperoxia.

A great portion of the studies in the literature on the prophylaxis of AFSC on pulmonary morbidities are those in which AFSC applications are done with extrinsic inoculation method. Our study, however, is one which sets forth in a natural medium the prophylactic effects of the markers and stem cells in AF against hyperoxia since it has been done on the rats in fetal period. Consequently, we are of the opinion that it has also revealed more rational data regarding the subject. The difference detected between the beneficial effects of AF on the pulmonary and hepatic tissues may have resulted from stem cells such as AFSC, AFMSC, which are contained in AF and have more dominant beneficial effects on the lung tissue [18,25,26].

Considering that premature deaths cannot globally be prevented and that the studies intended to reduce the morbidities, assumed to depend on oxygen in these newborns with AOx treatment modalities, continue progressively, our study has contributed to the studies aiming to reduce the morbidities in question, and presented a different perspective in regard to the subject for the authors conducting such studies.

## 5. Study limitations

Since tissue VEGF is a marker that must be studied together with the other markers following the removal of relevant tissues from the freezer and since our failure to obtain the VEGF kit involved in time, tissue VEGF values were kept out of our study. No doubt, if the tissue VEGF had been able to be studied, it would have been possible to interpret the effects of AF on tissue growth more consistently and this would have contributed to the scientific value of our study. We were able to include only 14 fetuses in our study. The reason for this was that only one fetus of a mother had to include in the study. In addition, the sex of these 14 fetuses could not be determined accurately, and therefore, they were not included in the assessment. The reason why we could not examine apoptosis with the Tunnel method was because we experienced technical problems and we wanted to attain the results rapidly. Nevertheless, we have planned to make a future study on this subject with Tunnel method and by including more samples tissue VEGF quantification.

## 6. Conclusion

Our study has demonstrated that in intrauterine period AF has important contributions to the protection of the pulmonary tissue from the toxic effects of oxygen and to the sustenance of normal tissue growth. In addition, the data we have obtained enabled us to suggest a new interpretation of the questions in the literature regarding the morbidities still continuing with a high incidence in our present day NICU’s and causing us to question the place of oxygen in the formation of postpartum morbidities in preterm infants. Besides all these, for a better understanding of the AF-Lung relationship, we should point out that there is a need for studies that will increase our knowledge of the literature and improve clinicians’ perspectives on the subject.

## Figures and Tables

**Table 1:** Oxidant and antioxidant marker values.

		NPRF (Mean ± SD) (n:7)	HPRF (Mean ± SD) (n: 7)	p value
**Lung tissue**	**tGSH (nmol/mL)**	12.22 ± 1.64	10.46 ± 0.99	**0.02**
	**CAT (U/mL)**	19.75 ± 4.13	15.75 ± 4.03	0.07
	**MDA (pmol/ml)**	2.05 ± 0.31	2.34 ± 0.21	**0.04**
	**TNF-α (pg/ml)**	2.46 ± 0.35	2.84 ± 0.44	0.07
**Liver tissue**	**tGSH (nmol/mL)**	14.29 ± 1.50	10.54 ± 1.44	**0.001**
	**CAT (U/mL)**	13.63 ± 2.92	10.38 ± 2.39	**0.03**
	**MDA (pmol/ml)**	1.81 ± 0.26	2.52 ± 0.49	**0.003**
	**TNF-α (pg/ml**)	3.88 ± 0.68	5.74 ± 0.91	**0.001**

**NPRF**: normoxic pregnant rat fetuses; **HPRF**: hyperoxic pregnant rat fetuses; **MDA**: malondialdehyde; **tGSH**: total glutathione; **CAT**: catalase; **TNF-α**: tumor necrosis factor alpha.

**Table 2:** Histopathology and histomorphometry values.

		NPRF (Mean ± SD) (n:7)	HPRF (Mean ± SD) (n:7)	p value
**Lung tissue**	**Apoptosis-caspase**	9.46 ± 2.86	11.66 ± 1.30	0.09
	**Apoptosis-HE**	6.57 ± 1.48	6.37 ± 0.69	0.75
	**AWC**	15.2 ± 1.6	15.2 ± 1.5	1.0
**Liver tissue**	**Apoptosis-caspase**	7.89 ± 1.2	12.2 ± 2.14	**0.001**
	**Apoptosis-HE**	2.71 ± 0.5	3.83 ± 1.04	**0.02**
	**VCC**	4.17 ± 0.3	4.26 ± 0.2	0.48

**NPRF**: normoxic pregnant rat fetuses; **HPRF**: hyperoxic pregnant rat fetuses; **HE**: hematoxylin-eosin; **VSC**: vena centralis count; **AWC**: alveolar wall count.

## References

[1] Escobar J Cernada M Vento M Oxygen and Oxidative Stress in the Neonatal Period Neoreviews 2011 26 12 613 624 10.1542/neo.12-11-e613

[2] Keszler M Sant’Anna G Mechanical Ventilation and Bronchopulmonary Dysplasia Clinics Perinatology 2015 42 4 781 796 10.1016/j.clp.2015.08.006 26593078

[3] Ben-Hur J Bernard T Bronchopulmonary dysplasia Polin Richard A MD Yoder Mervin C MD Workbook in Practical Neonatology 5th ed Philadelphia, PA, USA Elsevier Saunders 2015 169 182

[4] Friel JK Friesen RW Harding SV Roberts LJ Evidence of oxidative stress in full-term healthy infants Pediatric Research 2004 56 6 878 882 10.1203/01.PDR.0000146032.98120.43 15470194

[5] Maltepe E Saugstad OD Oxygen in health and disease: regulation of oxygen homeostasis; clinical implications Pediatric Research 2009 65 3 261 268 10.1203/PDR.0b013e31818fc83f 18852690

[6] Sultana Z Maiti K Aitken J Morris J Dedman L Oxidative stress, placental ageing-related pathologies and adverse pregnancy outcomes American Journal of Reproductive Immunology 2017 77 5 105 114 10.1111/aji.12653 28240397

[7] Novakovic TR Dolicanin ZC Djordjevic NZ Effects of maternal subclinical hypothyroidism on amniotic fluid cells oxidative status Reproductive Toxicology 2018 78 6 97 101 10.1016/j.reprotox.2018.04.002 29635049

[8] Tipple TE Ambalavanan N Oxygen Toxicity in the Neonate: Thinking Beyond the Balance Clinics Perinatology 2019 46 3 435 437 10.1016/j.clp.2019.05.001 PMC6662609 31345539

[9] Vento M Aguar M Escobar J Arduini A Escrig R Antenatal steroids and antioxidant enzyme activity in preterm infants: influence of gender and timing Antioxidants Redox Signaling 2009 11 12 2945 2955 10.1089/ars.2009.2671 19645572

[10] Forman HJ Fukuto JM Miller T Zhang H Rinna A The chemistry of cell signaling by reactive oxygen species and nitrogen species and 4-hydroxynonenal Archives of Biochemistry and Biophysics 2008 477 2 183 195 10.1016/j.abb.2008.06.011 18602883 PMC2590784

[11] Niedermaier S Hilgendorff A Bronchopulmonary dysplasia-an overview about pathophysiologic concepts Molecular and Cellular Pediatrics 2015 2 1 2 10.1186/s40348-015-0013-7 26542292 PMC4530566

[12] Saugstad OD Bronchopulmonary dysplasia-oxidative stress and antioxidants Seminars in Neonatology 2003 8 1 39 49 10.1016/s1084-2756(02)00194-x 12667829

[13] Schock BC Sweet DG Ennis M Warner JA Young IS Oxidative stress and increased type-IV collagenase levels in bronchoalveolar lavage fluid from newborn babies Pediatric Research 2001 50 1 29 33 10.1203/00006450-200107000-00008 11420415

[14] Moore TA Ahmad IM Schmid KK Berger AM Ruiz RJ Oxidative Stress Levels Throughout Pregnancy, at Birth, and in the Neonate Biological Research for Nursing 2019 21 5 485 494 10.1177/1099800419858670 31284724 PMC6854430

[15] Myatt L Review: Reactive oxygen and nitrogen species and functional adaptation of the placenta Placenta 2010 31 3 66 69 10.1016/j.placenta.2009.12.021 PMC2832707 20110125

[16] Smith LE Pathogenesis of retinopathy of prematurity Growth Hormone IGF Research 2004 14 6 140 144 10.1016/j.ghir.2004.03.030 15135797

[17] Fleck BW McIntosh N Retinopathy of prematurity: recent developments Neoreviews 2009 10 3 20 30 10.1542/neo.10-1-e20

[18] Grisafi D Pozzobon M Dedja A Vanzo V Tomanin R Human amniotic fluid stem cells protect rat lungs exposed to moderate hyperoxia Pediatric Pulmonology 2013 48 11 1070 1080 10.1002/ppul.22791 23533160

[19] Hodges RJ Jenkin G Hooper SB Allison B Lim R Human amnion epithelial cells reduce ventilation-induced preterm lung injury in fetal sheep American Journal of Obstetrics and Gynecology 2012 206 5 8 15 10.1016/j.ajog.2012.02.038 22542124

[20] Candilera V Bouchè C Schleef J Pederiva F Lung growth factors in the amniotic fluid of normal pregnancies and with congenital diaphragmatic hernia Journal Maternal Fetal Neonatal Medicine 2016 29 13 2104 2108 10.3109/14767058.2015.1076387 26333573

[21] Bedaiwy MA Burlingame JM Hussein M Flyckt R Assad R Assessment of vascular endothelial growth factor, basic fibroblast growth factor, and transforming growth factor levels in amniotic fluid The Journal of Reproductive Medicine 2012 57 10 405 410 23091987

[22] Porreco RP Bradshaw C Sarkar S Jones OW Enhanced Growth of amniotic fluid cells in presence of fibroblast growth factor Obstetrics & Gynecology 1980 55 1 55 59 7352062

[23] Gospodarowicz D Moran JS Owashi ND Effects of fibroblast growth factor and epidermal growth factor on the rate of growth of amniotic fluid-derived cells The Journal of Clinical Endocrinology and Metabolism 1977 44 4 651 659 10.1210/jcem-44-4-651 300379

[24] Da Sacco S De Filippo RE Perin L Amniotic fluid as a source of pluripotent and multipotent stem cells for organ regeneration Current Opinion in Organ Transplantation 2011 16 1 101 105 10.1097/MOT.0b013e3283424f6e 21157345

[25] Perin L Sedrakyan S Giuliani S Da Sacco S Carraro G Protective effect of human amniotic fluid stem cells in an immunodeficient mouse model of acute tubular necrosis PLOS One 2010 5 2 e9357 10.1371/journal.pone.0009357 20195358 PMC2827539

[26] Lan YW Yang JC Yen CC Huang TT Chen YC Predifferentiated amniotic fluid mesenchymal stem cells enhance lung alveolar epithelium regeneration and reverse elastase-induced pulmonary emphysema Stem Cell Research and Therapy 2019 10 1 163 171 10.1186/s13287-019-1282-1 31196196 PMC6567664

[27] Cheng FC Tai MH Sheu ML Chen CJ Yang DY Enhancement of regeneration with glia cell line-derived neurotrophic factor-transduced human amniotic fluid mesenchymal stem cells after sciatic nerve crush injury Journal of Neurosurgery 2010 112 4 868 879 10.3171/2009.8.JNS09850 19817545

[28] Carraro G Perin L Sedrakyan S Giuliani S Tiozzo C Human amniotic fluid stem cells can integrate and differentiate into epithelial lung lineages Stem Cells 2008 26 11 2902 2911 10.1634/stemcells.2008-0090 18719226 PMC3174105

[29] Murphy SV Atala A Amniotic fluid and placental membranes: unexpected sources of highly multipotent cells Seminars in Reproductive Medicine 2013 31 1 62 68 10.1055/s-0032-1331799 23329638

[30] Guven A Gundogdu G Uysal B Cermik H Kul M Hyperbaric oxygen therapy reduces the severity of necrotizing enterocolitis in a neonatal rat model Journal of Pediatric Surgery 2009 44 3 534 540 10.1016/j.jpedsurg.2008.06.008 19302854

[31] Sedlak J Lindsay RH Estimation of total, protein-bound, and nonprotein sulfhydryl groups in tissue with Ellman’s reagent Analytical Biochemistry 1968 25 7 192 205 10.1016/0003-2697(68)90092-4 4973948

[32] Ohkawa H Ohishi N Yagi K Assay for lipid peroxides in animal tissues by thiobarbituric acid reaction Analytical Biochemistry 1979 95 9 351 358 10.1016/0003-2697(79)90738-3 36810

[33] Ozdemir A Bastug O Cilenk KT Korkmaz L Korkut S et.al Can lycopene eliminate the harmful effects of hyperoxia in an immature brain? Archivos Argentinos de Pediatria 2019 117 4 237 244 10.5546/aap.2019.eng.237 31339266

[34] Bastug O Fatih Sonmez M Ozturk MA Korkmaz L Kesici H et. al Effects of Lycopene in Hyperoxia-Induced Lung Injury in Newborn Rats International Journal for Vitamin and Nutrition Research 2018 88 10 270 280 10.1024/0300-9831/a000238 31161929

[35] Ballard HO Bernard P Qualls J Everson W Shook LA Azithromycin protects against hyperoxic lung injury in neonatal rats Journal of Investigative Medicine 2007 55 11 299 305 10.2310/6650.2007.00011 17963679

[36] Steven H Abman MD Bronchopulmonary dysplasia Kending and Chernick’s Disorders of the Respiratory Tract in Children 8th ed Philadelphia, PA, USA Elsevier Saunders 2012 336 398

[37] Thannickal VJ Fanburg BL Reactive oxygen species in cell signaling American Journal of Physiology Lung Cellular and Molecular Physiology 2000 279 6 1005 1028 10.1152/ajplung.2000.279.6.L1005 11076791

[38] Lin PW Stoll BJ Necrotising enterocolitis Lancet 2006 368 9543 1271 1283 10.1016/S0140-6736(06)69525-1 17027734

[39] Welty SE Is Oxidant Stress in the Causal Pathway to Bronchopulmonary Dysplasia? NeoReviews 2000 1 1 6 10 10.1542/neo.1-1-e6

[40] Rabi Y Singhal N Nettel-Aguirre A Room-air versus oxygen administration for resuscitation of preterm infants: the ROAR study The Journal of Pediatrics 2011 128 2 374 381 10.1542/peds.2010-3130 21746729

[41] Finer NN Carlo WA Walsh MC Rich W Gantz MG SUPPORT Study Group of the Eunice Kennedy Shriver NICHD Neonatal Research Network Target ranges of oxygen saturation in extremely preterm infants The New England Journal of Medicine 2010 362 21 1970 1979 10.1056/NEJMoa0911781 20472937 PMC2891970

[42] Stenson BJ Tarnow-Mordi WO Darlow BA Simes J Juszczak E Oxygen saturation and outcomes in preterm infants The New England Journal of Medicine 2013 368 22 2094 2104 10.1056/NEJMoa1302298 23642047

[43] Taglauer E Abman SH Keller RL Recent advances in antenatal factors predisposing to bronchopulmonary dysplasia Seminars in Perinatology 2018 42 7 413 424 10.1053/j.semperi.2018.09.002 30389227 PMC6286866

[44] Rozance PJ Seedorf GJ Brown A Roe G O’Meara MC IUGR decreased pulmonary alveolar and vessel growth and causes pulmonary artery endothelial cell dysfunction in vitro in fetal sheep American Journal of Physiology 2011 301 6 860 871 10.1152/ajplung.00197.2011 PMC3233832 21873446

[45] Sun L Marti HH Veltkamp R Hyperbaric oxygen reduces tissue hypoxia and hypoxia-inducible factor-1 alpha expression in focal cerebral ischemia Stroke 2008 39 3 1000 1006 10.1161/STROKEAHA.107.490599 18239183

[46] Novakovic TR Dolicanin ZC Djordjevic NZ Oxidative stress biomarkers in amniotic fluid of pregnant women with hypothyroidism The Journal of Maternal Fetal and Neonatal Medicine 2019 32 7 1105 1110 10.1080/14767058.2017.1400005 29141467

[47] Mustafa MD Pathak R Ahmed T Ahmed RS Tripathi AK Association of glutathione S-transferase M1 and T1 gene polymorphisms and oxidative stress markers in preterm labor Clinical Biochemistry 2010 43 13–14 1124 1128 10.1016/j.clinbiochem.2010.06.018 20621079

[48] Cinkaya A Keskin HL Buyukkagnici U Gungor T Keskin EA Maternal plasma total antioxidant status in preterm labor Journal of Obstetrics and Gynaecology Research 2010 36 6 1185 1188 10.1111/j.1447-0756.2010.01300.x 21040202

[49] Mei Y Chen C Dong H Zhang W Wang Y Treatment of Hyperoxia-Induced Lung Injury with Lung Mesenchymal Stem Cells in Mice Stem Cells International 2018 2018 5976519 10.1155/2018/5976519 30356447 PMC6178508

[50] Aceti A Beghetti I Martini S Faldella G Corvaglia L Oxidative Stress and Necrotizing Enterocolitis: Pathogenetic Mechanisms, Opportunities for Intervention, and Role of Human Milk Oxidative Medicine and Cellular Longevity 2018 2 7 7397659 10.1155/2018/7397659 PMC6051049 30057683

[51] Oter S Korkmaz A Topal T Ozcan O Sadir S Correlation between hyperbaric oxygen exposure pressures and oxidative parameters in rat lung, brain, and erythrocytes Clinical Biochemistry 2005 38 8 706 711 10.1016/j.clinbiochem.2005.04.005 15904909

